# Role of previous hospitalization in clinically-significant MRSA infection among HIV-infected inpatients: results of a case-control study

**DOI:** 10.1186/1471-2334-7-36

**Published:** 2007-04-30

**Authors:** Cecilia MJ Drapeau, Claudio Angeletti, Anna Festa, Nicola Petrosillo

**Affiliations:** 1 2^nd ^Infectious Diseases Division, National Institute for Infectious Diseases "L. Spallanzani", Rome, Italy; 2 Epidemiology Department, National Institute for Infectious Diseases "L. Spallanzani", Rome, Italy; 3 Microbiology Laboratory, National Institute for Infectious Diseases "L. Spallanzani", Rome, Italy

## Abstract

**Background:**

HIV-infected subjects have high incidence rates of *Staphylococcus aureus *infections, with both methicillin-susceptible and methicillin-resistant (MRSA) strains. Possible explanations could include the high burden of colonization, the behavioral risk factors, and the frequent exposures to health care facilities of HIV-infected patients. The purpose of the study was to assess the risk factors for clinically- significant methicillin-resistant *Staphylococcus aureus *(CS-MRSA) infections in HIV-infected patients admitted to Infectious Diseases Units.

**Methods:**

From January 1, 2002 to December 31, 2005, we conducted a retrospective case-control (1:2) study. We identified all the cases of CS-MRSA infections in HIV-infected patients admitted to the National Institute for Infectious Diseases (INMI) "Lazzaro Spallanzani" in the 4-year study period. A conditional logistic regression model was used to identify risk factors for CS-MRSA infection.

**Results:**

We found 27 CS-MRSA infections, i.e. 0.9 CS-MRSA infections per 100 HIV-infected individuals cared for in our Institute. At multivariate analysis, independent predictors of CS-MRSA infection were cumulative hospital stay, invasive procedures in the previous year, and low CD4 cell count. Particularly, the risk for CS-MRSA increased by 14% per an increase of 5 days hospitalization in the previous year. Finally, we identified a low frequency of community-acquired MRSA infections (only 1 of 27; 3.7%) among HIV-infected patients.

**Conclusion:**

Clinicians should be aware of the risk for CS-MRSA infection in the clinical management of HIV-infected patients, especially in those patients with a low CD4 cell count, longer previous hospital stay, and previous invasive procedures.

## Background

According to the European Antimicrobial Resistance Surveillance System 2004 report, the overall prevalence of methicillin-resistant S. *aureus *(MRSA) isolates in European hospitals is around 24% [1], and Italy is considered to be one of the highest incidence countries (about 50%) [1]. MRSA infections represent a substantial burden in terms of morbidity, mortality, and costs [2]. Established risk factors for health care-associated MRSA (HC-MRSA) infections include recent hospitalization or surgery, residence in a long-term care facility, dialysis, indwelling percutaneous devices and catheters [3,4]. Moreover, in the last decades, several cases of MRSA infections without established risk factors for health care-associated acquisition have been documented and referred to as community-associated MRSA (CA-MRSA) infections [5].

HIV-infected subjects have high incidence rates of *S. aureus *infections [6-10] probably due to the high burden of colonization [8,11-14], to behavioral risk factors [15] and to frequent health care facility exposures [9,16].

We have retrospectively reviewed the cases of CS-MRSA infections occurring among HIV-infected patients admitted to our Infectious Diseases hospital, with the aim to evaluate the clinical and epidemiological associated risk factors.

## Methods

We conducted a retrospective case-control study on HIV-infected patients admitted to our Institute for Infectious Diseases "L. Spallanzani, Rome from January 1, 2002 to December 31, 2005. Approximately 3,000 HIV-infected patients were followed up in our outpatient facility in the 4-year study period. We collected all the microbiological reports yielding MRSA isolates from different body sites except stool with their respective antibiotic sensitivity tests reported at the computer database of our Institute's microbiological laboratory. Microbiological cultures were taken before empirical antibiotic treatment. Surveillance cultures for colonization were excluded from the study. Sensitivity testing at our laboratory was performed using Phoenix (Becton Dickinson) automated system. MRSA was defined as minimum inhibiting concentration ≥ 4 micrograms/milliliter for oxacillin.

Medical records were reviewed to identify those patients who had a documented clinically-significant MRSA (CS-MRSA) infection at isolation time. The clinical significance of each MRSA isolate was settled on one or more of the following criteria: 1) a positive MRSA culture from a normally sterile body site; 2) documented clinical diagnosis of infection at isolation time (e.g. sputum isolates in patients with documented pneumonia). Moreover, information about the patient's demographic and clinical features (sex, age, HIV risk factor) was ascertained. The MRSA isolates were classified according to the source of isolation, as follows: 1) skin, soft tissue isolates; 2) respiratory isolates including sputum, bronchoalveolar lavage fluid and pleural fluid; 3) blood isolates.

Further investigation was conducted through the hospital computer database and the past medical records review, to assess the following data concerning the patient's immunological, clinical and hospitalization history prior to CS-MRSA infection onset: 1) median CD4 cell count during past half year; 2) median log_10 _HIV plasma viral load during past half year; 3) HIV clinical group according to Centers for Disease Control and Prevention (CDC), Atlanta; 4) any antiretroviral (ARV) therapy during past half year; 5) any cotrimoxazole prophylaxis in the previous 3 months; 6) peritoneal or haemodialysis or 7) surgery within 12 months before the MRSA infection; 8) presence of a percutaneous device or indwelling vascular catheter at the time of infection or placed in the past 12 months; 9) total number of hospital days and 10) total number of clinical visits to our outpatient facility in the previous year. Time period for ARV therapy and cotrimoxazole prophylaxis was arbitrarily selected according to previous studies [15].

CS-MRSA infection was defined either health care or community-associated, based on established criteria for definition of "community-associated MRSA infection" [17]. In particular, infection was considered as health care-associated if the patient had a clinical history of the following risk factors: MRSA infection identified after 48 hours of admission to hospital; a history of hospitalization, surgery, dialysis or residence in long-term care facility within one year of the MRSA culture date; a permanent indwelling catheter or percutaneous medical device present at the time of culture or a known positive culture for MRSA prior to the study period. Lack of the above health care risk factors led to community-associated MRSA infection case definition.

A retrospective case control-study was performed, considering as cases those HIV-infected patients with MRSA infection identified during the hospital stay, and as controls those HIV-infected patients without MRSA infection, admitted to the same wards in the study period. Controls were randomized 1:2 and matched by sex and age.

To assess potential risk factors for CS-MRSA infection, we used a conditional logistic regression model and results were exposed in terms of Odds Ratio (OR) with their respective 95% Confidence Interval (CI). We performed a univariate analysis by adjusting each variable for age at enrolment (< = 42 years and >42 years of age), in order to avoid any residual confounding due to the matching of the variables. All the covariates that were found to be significantly associated (p < 0.05) with CS-MRSA infection were included in a multivariate model, and afterwards, using a stepwise backward procedure, we selected the final model that included the following variables: age at the enrollment, total number of hospital days in the previous year (modeled as continuous variable), number of outpatient visits in the 12 months prior to MRSA diagnosis (0–1 visit and more than 1 visit), any submission to invasive procedures (including percutaneous device or indwelling vascular catheter present at the time of infection or placed in the past 12 months, any peritoneal or haemodialysis, and surgical procedures within the past year), and the median CD4 cell count in the previous six months. We also plotted the trend of OR according to total number of hospital days in the previous year, by dividing the patients with at least one day of hospitalization in three groups of approximately the same number of days (0, 1–12, 14–45 and >45 days), and performed a test to verify its significance.

Since the data reported in our study was collected by patient's chart review, and no additional information or any other intervention was made for the purpose of the study, no specific ethical approval was requested according to the regulations of our institute.

## Results

During the 4-year study period, 5,257 admissions of HIV-infected patients occurred at our Institute for Infectious Diseases "Lazzaro Spallanzani", Rome, Italy. The microbiological computer database found out a total of 28 HIV-infected patients yielding positive MRSA cultures; 27 patients had CS-MRSA infection, and accounted for 0.5% of total admissions of HIV-infected patients, and for 0.9% of total HIV-infected patients. Males were 20 (74%); median age was 43 years (range 38–48). Twenty out of them (74%) had median CD4 < 200/microliter (μl), and 9 (34.6%) had median log_10 _HIV plasma viral load ≤ 2.99 copies/milliliter (cp/ml). Four (14.8%) and 23 (85.2%) patients were classified as HIV group B, and C, respectively. Fifty-four controls were enrolled; males were 40 (74%), median age was 44 years (range 37–48). Thirty-two patients (59.2%) had CD4 ≥ 200/μl, 14 patients (26%) had median log_10 _HIV plasma viral load ≤ 2.99 cp/ml. Four (7.4%), 22 (40.7%), and 28 (51.8%) patients were respectively classified as CDC group A, B, and C. No significant differences were found according to HIV risk factors; particularly drug users were 55.6% among cases and 57.3% among controls.

Of the 27 HIV-infected patients, primary sources of MRSA were distributed as follows: 11 (40.8%) blood, 8 (29.6%) skin and soft tissue, and 8 (29.6%) respiratory (1 pleural fluid, 2 bronchoalveolar fluid lavage, 5 sputum).

Among the 27 patients with MRSA infection, 17 (63%) had a MRSA infection identified within 48 hours of admission (7 had pneumonia, 6 had skin or soft tissue infections, 4 had sepsis); 10 (37%) patients evidenced MRSA infection during the hospital stay (8 had sepsis of which 2 were indwelling vascular device-related, 2 had skin and soft tissue infections). According to the above mentioned criteria, twenty-six out of 27 patients had health care-associated, and only 1 had community-associated MRSA infection. Neither cases nor controls were involved in any case clustering or outbreak during the study period.

**Figure 1:**
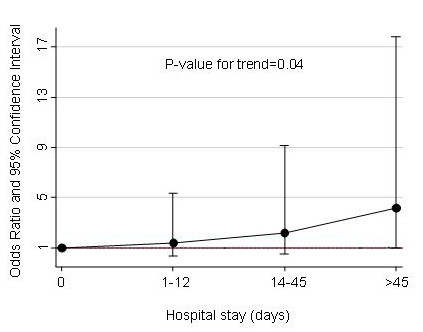
**Odds Ratios of MRSA infection according to cumulative hospital stay in the previous year**. Trend of Odd Ratios of MRSA infection adjusted for age (≤ 42 years and > 42 years) according to total number of hospital days in the previous year.

Univariate analysis revealed that the following variables were associated with the occurrence of CS-MRSA infection: longer cumulative hospital stay in the previous year, less than 2 outpatient visit in the previous year, invasive procedures in the previous year, and CD4 cell count < 200/μl. Moreover, the administration of cotrimoxazole prophylaxis in the previous 4 months was more common among cases (29.6%) than controls (9.3%), and close to significancy (p = 0.051). At multivariate analysis, cumulative hospital stay, invasive procedures in the previous year, and CD4 cell count in the previous 6 months remained associated with CS-MRSA infection (Table 1). Of note, the risk for CS-MRSA increased by 14% per an increase of 5 days hospitalization. The odds ratios of CS-MRSA infection according to cumulative hospital stay in the previous year (p-value for trend = 0.04) are reported in Figure 1. Mortality rate was 6/27 (22%) among cases and 6/54 (11%) among controls (OR 2.28; 95% CI = 0.66–7.91; p = 0.10).

**Table 1:** Risk factors for CS-MRSA^ infection in HIV infected patients. Multivariate analysis.

	**Cases n = 27**	**Controls n = 54**	**Univariate analysis**				**Multivariate analysis**			
			**Odds Ratio§**	**95% CI^^**		p*	**Odds Ratio§**	**95% CI^^**		p*

**HIV risk factor, n (%)**										
IDU (intravenous drug user)	15	31	1.00	-	-	-	-	-	-	-
Homosexual	2	7	0.50	0.08	3.02	0.449	-	-	-	-
Heterosexual	5	7	1.70	0.41	6.97	0.463	-	-	-	-
Unknown	5	9	1.18	0.33	4.22	0.801	-	-	-	-
**Cumulative hospital stay (days) in the previous year**										
(per an increase of 5 days)	-	-	1.11	1.02	1.22	0.020	1.14	1.01	1.29	0.041
**Outpatient visits in the previous year**										
0–1	17	19	1.00	-	-	-	1.00	-	-	-
more than 1	10	35	0.34	0.13	0.92	0.034	0.26	0.07	1.01	0.051
Any invasive procedure in the previous year**										
No	17	51	1.00	-	-	-	1.00	-	-	-
Yes	10	3	8.92	1.93	41.31	0.005	9.14	1.31	63.73	0.025
**Median CD4 cell count previous half year (cells/μl)**										
0–199	20	22	1.00	-	-	-	1.00	-	-	-
≥ 200	7	32	0.24	0.08	0.73	0.013	0.22	0.05	1.00	0.050
**Median Log**_**10**_HIV plasma viral load (cp/ml) in the previous half year***										
≤ 2.99	9	14	1.00	-	-	-	-	-	-	-
≥ 3	17	39	0.69	0.25	1.92	0.478	-	-	-	-
**Any ARV therapy in previous 6 months**										
No	17	41	1.00	-	-	-	-	-	-	-
Yes	10	13	1.86	0.65	5.29	0.245	-	-	-	-
**Any cotrimoxazole in the previous 4 months**										
No	19	49	1.00	-	-	-	-	-	-	-
Yes	8	5	3.06	0.99	9.41	0.051	-	-	-	-

^ CS = clinically significant methicillin-resistant *Staphylococcus aureus*

^^ 95% confidence intervals

§ All Estimates are adjusted for age at enrolment (< = 42 years and >42 years of age)

* Wald test

** It Includes surgery, dialysis or a permanent indwelling catheter or percutaneous medical device.

*** Only 77 patients considered; 2 patients not added up to total for missing values.

## Discussion

In our 4-year study, we found a 0.5 rate of CS-MRSA infections per 100 admissions of HIV-infected patients, and an average 0.9 CS-MRSA infections per 100 HIV-infected patients followed up in our outpatient facility. The MRSA infection rate we found among HIV-infected patients is slightly lower than those reported in the literature (range 1.9%–3%) [15,16]. Possible explanations could include the features of the study population (i.e. jail inmates), [16] and the differences in MRSA clinically-significant infection definition [16].

In our study we identified a low frequency of CA-MRSA infection among HIV-infected patients. Indeed, only 1 out of 27 MRSA patients (3.7%) fulfilled CA-MRSA definition [17]. This patient was an intravenous drug user who was admitted to hospital with a diagnosis of cellulitis of the inferior limb; he had a median CD4 cell count lower than 200/μl, and a median log_10 _HIV plasma viral load > 2.99 cp/ml. Our findings are in contrast with those of Matthews et al. [15] that reported a 60% of community-associated CS-MRSA infections among HIV-infected patients. However, in a recent study conducted by Seybold et al [18], 9 out of 116 MRSA bloodstream infections (8%), of which 22% occurring in HIV-infected patients, were community-associated. In another study conducted by Naimi et al. [19], among non HIV-infected patients, 131 out of 1,100 MRSA infections (12%) were community-associated.

To our knowledge, to date in the literature, only few cases of CA-MRSA infections have been described in Italy [20,21] and in only one case, i.e. in a non HIV-infected patient, there was evidence of Panton-Valentine leukocidin production [20].

In our retrospective study we found that length of cumulative hospital stay, submission to invasive procedures in the 12 months prior to MRSA infection onset, and low CD4 cell count were variables independently associated with CS-MRSA infection. These findings are in agreement with previous reports of the literature [9,15,16,22]. Moreover, only in another study [16], the authors found that total hospital days in the preceding six months were independently associated with an increased risk for MRSA colonization and infection in HIV-infected patients. The finding that MRSA infection is related to previous hospitalization has been reported in different populations, but actually only few studies have focused on HIV population. Interestingly, in our study we were also able to quantify the risk of CS-MRSA infection according to the increase of hospital stay in the previous year, i.e. 14% per an increase of 5 days (Figure 1).

In our study we also found that attendance to our outpatient clinic was less frequent in MRSA cases than controls. Possible explanations to this finding include the low invasive procedure rate in the HIV outpatient setting in our country, resulting in a low exposure rate to nosocomial organisms; moreover, the higher CD4 cell count we found among controls may be a protective factor for MRSA colonization according to data reported in literature,[14,23] and for the occurrence of opportunistic infections that lead to hospitalization. In consequence, controls are likely to have more clinical visits in outpatient settings than cases.

Most of our HC-MRSA infections (63%) had a community onset, not far from the 50% rate found by Seybold et al. among 107 HC-MRSA [14]. Moreover, we found that about 40% cases had MRSA sepsis and 30% had skin, and soft tissue infections. Several reports have pointed out on the higher prevalence of MRSA bloodstream and skin and soft tissue infections among HIV-infected patients with respect to other MRSA clinical infections [9,15,16]. The relatively high rate of MRSA sepsis could in part explain why our cases had a two-fold mortality rate than controls.

Our study had some limitations, including the limited number of cases; however, in order to increase the potency of our study we enrolled two controls per each case. Furthermore, owing to the retrospective design of our study, no information is available on previous wide-spectrum antibiotic treatments, and on other potential risk factors reported in the literature [12,13]. We may also have underestimated the total number of hospital days in the previous year as we did not consider hospitalization in other hospitals. We believe this information to be of unremarkable weight because in our country, HIV-infected inpatients refer only to Infectious Diseases wards for medical acute care, and our Institute is a referral center for HIV-infected patients in our region. Consequently, it is likely that almost all cases of CS-MRSA infections occurring in our HIV-infected outpatient population were admitted to our Institute.

Finally, we are aware that the total number of CS-MRSA infection diagnosis at our Institute especially in the outpatient settings could have been underestimated because of the following reasons: 1) our isolates were collected during hospital stay whereas in other studies, MRSA isolates were collected also during outpatient visits [15,16]; 2) some clinically diagnosed mild infections that do not require hospitalization could have been treated with empirical antibiotic therapy, leading to misdiagnosis of MRSA infection and/or to culture-negative samples when collected.

## Conclusion

In conclusion, our study represents an attempt to evaluate the potential clinical conditions associated with a CS-MRSA infection among HIV-infected patient; as recently proposed by Harbarth et al, these findings could be used to calculate a risk score at admission [24].

In the clinical management of HIV-infected patients, especially in those admitted to hospital for clinical sepsis, and/or skin and soft tissue infections, clinicians should be aware of the risk factors associated with a MRSA infection, particularly low CD4 cell count, longer previous hospital stay and previous invasive procedures.

## Competing interests

Financial competing interests: NP is on the speakers' bureau for several companies including Merck Sharpe & Dohme, Aventis, Ethicon, GSK, Pfizer, AstraZeneca, Roche, Gilead. None of the other authors has financial competing interests.

None of the authors has non financial competing interests to disclose.

## Authors' contributions

CMJD contributed to the study design, to the acquisition of clinical and microbiological data and to manuscript draft and final version.

CA performed statistical analysis, and contributed to interpretation of data.

AF contributed to acquisition of microbiological data, and to the manuscript draft.

NP conceived the study, contributed to its design, and to the manuscript draft and final version.

All authors read and approved the final manuscript.

## Pre-publication history

The pre-publication history for this paper can be accessed here:


